# Extraretinal Induced Visual Sensations during IMRT of the Brain

**DOI:** 10.1371/journal.pone.0123440

**Published:** 2015-04-15

**Authors:** Timo Wilhelm-Buchstab, Barbara Myrthe Buchstab, Christina Leitzen, Stephan Garbe, Thomas Müdder, Susanne Oberste-Beulmann, Alois Martin Sprinkart, Birgit Simon, Michael Nelles, Wolfgang Block, Felix Schoroth, Hans Heinz Schild, Heinrich Schüller

**Affiliations:** University of Bonn, Department of Radiology, Radiooncology, Sigmund-Freud-Straße 25, 53105, Bonn, Deutschland, Germany; NIH, UNITED STATES

## Abstract

**Background:**

We observed visual sensations (VSs) in patients undergoing intensity modulated radiotherapy (IMRT) of the brain without the beam passing through ocular structures. We analyzed this phenomenon especially with regards to reproducibility, and origin.

**Methods and Findings:**

Analyzed were ten consecutive patients (aged 41-71 years) with glioblastoma multiforme who received pulsed IMRT (total dose 60Gy) with helical tomotherapy (TT). A megavolt—CT (MVCT) was performed daily before treatment. VSs were reported and recorded using a triggered event recorder. The frequency of VSs was calculated and VSs were correlated with beam direction and couch position. Subjective patient perception was plotted on an 8x8 visual field (VF) matrix. Distance to the orbital roof (OR) from the first beam causing a VS was calculated from the Dicom radiation therapy data and MVCT data. During 175 treatment sessions (average 17.5 per patient) 5959 VSs were recorded and analyzed. VSs occurred only during the treatment session not during the MVCTs. Plotting events over time revealed patient-specific patterns. The average cranio-caudad extension of VS-inducing area was 63.4mm (range 43.24-92.1mm). The maximum distance between the first VS and the OR was 56.1mm so that direct interaction with the retina is unlikely. Data on subjective visual perception showed that VSs occurred mainly in the upper right and left quadrants of the VF. Within the visual pathways the highest probability for origin of VSs was seen in the optic chiasm and the optic tract (22%).

**Conclusions:**

There is clear evidence that interaction of photon irradiation with neuronal structures distant from the eye can lead to VSs.

## Introduction

There is a long history of reports of visual sensations (VSs) caused by radiation, under various circumstances [1–4]. For example, VSs were reported by astronauts during the Apollo mission space flights [5–11]; it was suggested that Cherenkov radiation [12] or direct interaction of radiation particles, as protons, heavy ions or neutrons with the retina were responsible [13–21]. Even though one might have the impression that occurrence of VSs is completely understood, especially with regards to their origin, this is not the case. We observed VSs in a patient undergoing stereotactic IMRT for a single brain metastasis under circumstances in which direct interaction with the retina was improbable [22]. This study was intended to further elucidate this finding and test the hypothesis that radiation- induced VS may be of extraocular origin.

## Methods

The study protocol was approved by the local ethical review committee: Ethikkommission der Rheinischen Friedrich Wilhelm-Universität Bonn. All participants had to pass a consent procedure and had to provide their written declaration of consent (approved by the ethical review committee) before they were included in this study.

The study included 10 patients, all of whom had reported light flashes during their first radiation treatment. All patients suffered from WHO Grade IV glioma and received image guided helical intensity modulated radiotherapy (IMRT) with a Tomotherapy Hi Art System (TT). The 3 women and 7 men were between 41 and 71 years of age. Nine of the ten patients underwent adjuvant combined radio-chemotherapy (Temozolomid or Bevacizumab) after surgery; one received primary radio-chemotherapy. Five patients were on anticonvulsive medication (Levetirazepam). All patients were positioned on a positioning board (All in one, Orfit) in a supine position for radiation treatment. A three-point thermoplastic head fixation mask (Orfit) was used to ensure that the head was positioned in a stable and comfortable position. Daily Megavoltage Computed Tomography (MVCT) was matched to the planning computed tomography, and the table was adjusted manually to ensure that the patient was positioned correctly before irradiation. Radiotherapy was delivered using a 25 mm (n = 9) or 10 mm (n = 1) beam size with a pitch of 0.43. Patients received a total dose of 60 Gy in daily fractions of 2Gy.

Patients were allowed to keep their eyes open or closed during treatment, and their choice was recorded.

### Event registration and analysis

An event recorder was placed in the right hand of the patients and they were instructed to press a push button whenever they experienced a VS. Events were recorded with a digital audio editor (audacity 1.2.6) during all treatment sessions i.e. during radiation therapy and during the preceding MVCT.

After radiation treatment the patients were asked to describe their visual sensations in detail (color, shape, brightness, intensity etc.) and indicate their location on a visual field diagram.

Information on beam angle, leaf opening time and table position was extracted from the radiation treatment planning (RTP) file. This allowed us to analyze the beam characteristics, specifically the beam path, at the times VSs were recorded. The orbital roof was used as a reference point. To make a distinction between a radiation beam which could affect the optic pathways (or not) in relation to bony anatomic structures a point in a distance of half a beam width above the highest point of the orbital roof was found best in all patients. We were able to differentiate VSs caused by supraorbital beam paths from events which occurred when the orbits and optic pathways were in line with the path of the treatment beam.

Converting Dicom (Digital Imaging and communications in Medicine) data to NIfTI (Neuroimaging Informatics Technology Initiative) format transformation to standardized brain magnetic resonance tomography (MRI) images was possible by using mricron (version 6/2013, NITRC, Chris Rorden) and evildicom library (Rex Cardan, version 0.0.5.7, http://www.rexcardan.com/evildicom/). Using ANTS (Advanced normalization tools software by PICSL (Penn Image Computing & Science Lab) affine transformation of planning CT to standardized MRI was possible. As we observed that VSs occurred only during treatment mode (and not during the preceding CT, see below) we defined a threshold to eliminate beams which deposit a dose > 1% to the retina; in this way all beams closer than 40 mm to the eyeballs were eliminated.

Furthermore we correlated beam path with the incidence of VSs, and evaluated their frequency along the extra- and intracerebral visual pathways. All parts of the visual apparatus, including eyes, optic nerves, chiasma, optical tract, optic radiation and Brodman areas 17, 18, 19, 20, 21, 31 were defined in standardized MRI image, and a probability estimation for the origin of the VSs was performed.

## Results

All patients who had reported VSs during their first treatment session (the inclusion criterion) also reported VSs during the subsequent 29 treatments.

There were no reports of VSs during daily pre-treatment MVCT scans.

Data from 175/290 (60.3%) treatment fractions were analyzed; data from 115 treatment sessions were discarded for various technical reasons such as malfunction of the event recorder, broken cables etc.

5959 VSs were registered during the 175 analyzed sessions.

In total, we analyzed 97285 different beams, on average 580 (359–877) beams per session (Table 1).

**Table 1:** Beam path an occurrence of visual sensations in relation to bony structure.

Patient	Beams/fraction	fractions	VSs	VSs above the orbital roof	VSs below the orbital roof
1	472	17	74	29	45
2	637	20	343	198	145
3	820	7	530	232	298
4	574	16	99	54	45
5	435	13	113	1	112
6	458	17	1481	592	889
7	877	22	950	514	436
8	688	26	1132	622	510
9	478	15	478	143	335
10	359	22	759	585	174
Sum		175	5959	2970	2989

During 175 treatment fractions 97285 beams were administered. 5959 visual sensations were registered. To exclude direct interaction with the retina all supraorbital VSs caused by beams with central beam axis distance < ½ beam size to the orbital roof were eliminated. From this 2970 (50%) VSs occurred above the orbital roof without direct interaction to the retina.

**Fig 1:**
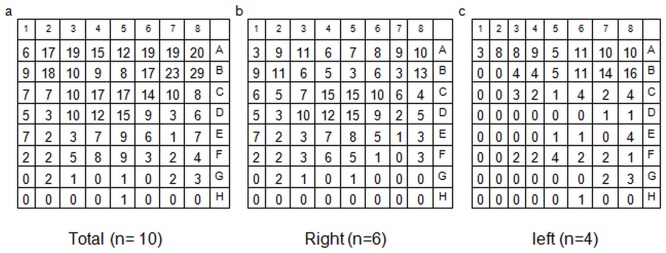
Analysis of the 8x8 visual field matrix of all 10 Patients (100 fractions). Summation of all perceptions independent from primary tumor site (a). Perception of visual sensations divided in patients with right (b) and left sided tumor location (c). Visual sensations were mainly projected in the upper right or left and the central visual field but not in the lower parts suitable for the majority of the beams. No relationship between primary tumor site and location of light perception can be made. Interestingly most beams were located supraorbital. However the main VSs were registered in the upper visual field and not, as expected in the lower quadrants.

Patients reported VSs of white and blue colored light flashes, or arc-shaped light formations, sometimes moving from left to right, or right to left. Three patients described vessel-like light contours. Analysis of the 8x8 visual field matrix demonstrated that VS were mainly assigned to the upper left or right visual fields (Fig 1).

**Fig 2:**
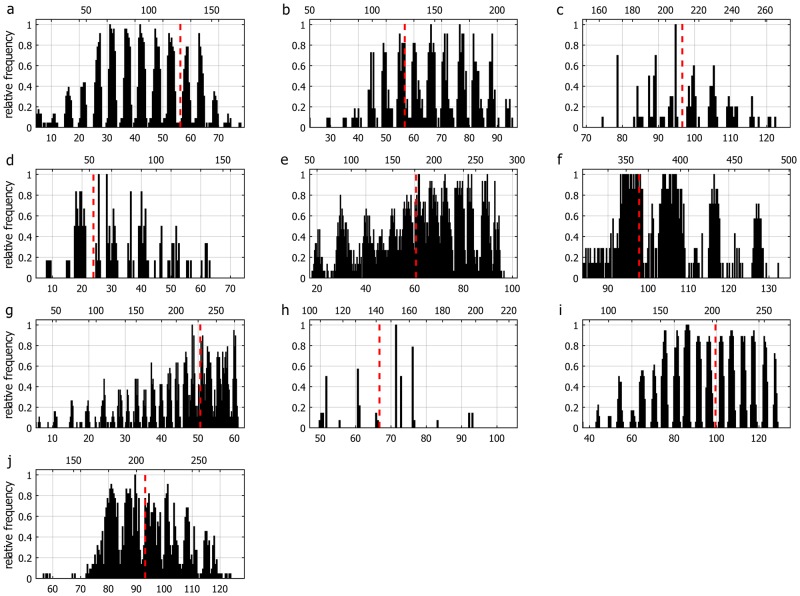
Diagram of the registered data of all patients 1–10 (a-j) shows patient specific patterns of relative frequency of the visual sensations (1 = light sensation was registered in all fractions) related to cranio-caudal couch travel [mm] (lower horizontal axis) and fraction time [s] (upper horizontal axis). Position of orbital roof is marked with a red dashed line. Every patient showed a pattern, which indicates that the VSs are induced by specific beam paths.

**Fig 3:**
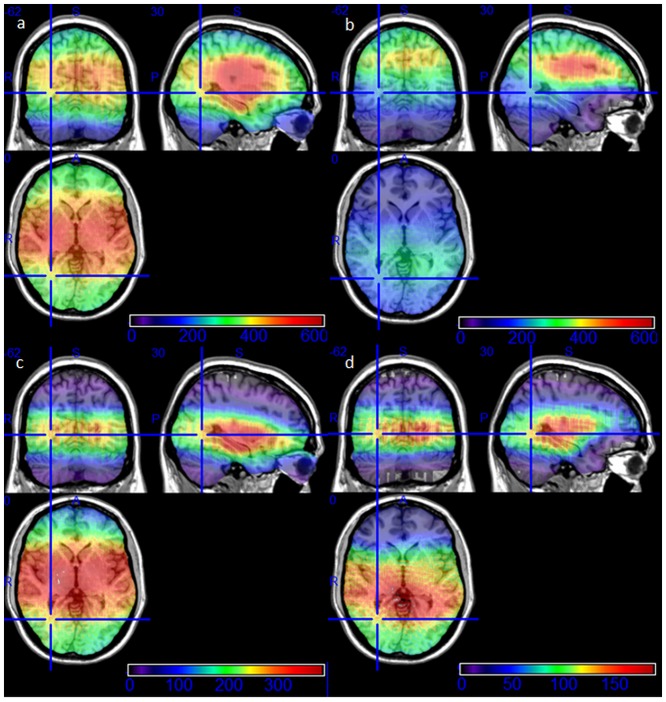
Anatomic correlation of the treatment beams using standardized MRI image series. The different frequency can be seen in the colored bar. (a) Exposed brain areas during all 175 treatment sessions. Most beams were located in the center of the brain. (b) All beams passing the eyeballs in a distance > 4cm, so that direct beam path did not hit the eyeballs. (c) All beams which induced a visual sensation. Highest frequency is seen in the mid brain and temporal lobe, less in the occipital lobe, least in the parietal brain areas. (d) All beams passing the eyes in a distance > 4 cm inducing a visual sensation. Direct interaction of radiation with the retina is impossible for these visual perceptions. This anatomic correlation shows a high frequency along the intracerebral visual pathways.

The VSs were recorded in all subsequent evaluable treatment sessions by all patients and occurred regardless of whether the patient’s eyes were open or shut. Although the reported intensity was lower when eyes were open. The correlation of the events and the beam paths revealed that 5.9% of all beams (5959 of 97285 beams) induced VSs. 4% (2970 of 74460 beams) of supraorbital beam paths induced VSs, while beams below the orbital roof induced VSs in 13,1% (2989/22825). In any given patient the beam path parameters associated with VSs were remarkably constant. There were patient-specific patterns associated with occurrence and absence of VSs. These patterns were consistent over all treatment sessions (Fig 2). The elimination of all beams causing VSs passing the eyes closer than 40 mm allowed us to discriminate intracerebral regions along the optical tract which seem to be responsible for VSs other than orbital structures (Fig 3).

Anatomic correlation of all beam paths affecting the visual pathways with the incidence of VSs revealed probabilities for being the origin of VSs as demonstrated in Table 2.

**Table 2:** Probabilities of anatomic structures along the optic pathway for being the origin of radiation induced visual sensations.

VOI	Number of beams				Beams inducing visual sensations			
	Average	Sigma	Min	Max	Average [%]	Sigma	Min	Max
EB	110.6	39.5	24	208	10.1	5.1	1	26
ON (intra)	168.5	40	85	277	15.4	4.1	6	25
ON (retro)CH, OT	344.5	66.7	199	542	22.3	1.9	14	27
Brodman areas								
A17	419.3	63.3	282	611	14.8	2.1	6	20
A18	390.5	55.1	222	577	12.6	3.6	1	22
A19	398.2	55.3	219	590	11.2	5.2	1	21
A20	400.5	86.1	158	606	17.5	1.8	11	23
A21	476.4	54.7	260	609	17.7	2.6	6	25
A37	420.1	66.4	207	609	16.1	2	7	22

Volume of interest (VOI), including the eyeballs (EB), optic nerve intraorbital (ON intra), optic nerve retroorbital (ON retro), chiasma (CH), optic tract (OT) and Brodman Areas A17, A18, A19, A20, A21 and A37, showing different probability for being the origin of the visual sensations based on the frequency of light perception, when they are hit by a beam. Interestingly the probability increases in retroorbital structures, especially optic chiasm, optic tract and in the brodman areas 20, 21, 37. The eye balls showed lowest probability of all VOIs.

We evaluated the doses in the extraretinal visual pathways (optic chiasm and optic nerve) which showed highest probability for being the origin of the VSs (Table 3). The maximum shielding of the eyeballs resulted in low doses in the optical nerves and an increased dose in the optic chiasm.

**Table 3:** Dose distribution in organs at risk.

	Optic chiasm			Optic nerve left			Optic nerve right		
Pat.	min	max	mean	min	max	mean	min	max	mean
1	15.20	39.52	18.69	6.05	63.44	14.25	5.68	25.65	20.40
2	9.60	30.92	16.01	10.97	34.42	18.82	8.41	12.84	10.13
3	6.00	35.44	13.09	5.99	14.94	9.56	4.14	30.37	16.32
4	27.60	42.90	35.83	14.00	46.24	35.76	13.74	38.00	28.49
5	28.20	58.56	51.66	12.42	54.43	46.18	11.63	32.38	24.78
6	18.25	50.36	25.27	8.59	27.49	20.71	7.17	46.39	23.03
7	7.35	46.93	21.43	5.13	11.48	8.56	4.30	25.31	11.75
8	29.77	33.98	32.35	9.47	28.88	14.78	8.17	30.23	12.60
9	5.73	26.62	9.26	7.42	23.79	12.71	4.29	8.20	5.39
10	2.04	32.39	5.09	0.94	4.55	1.35	0.88	3.31	1.19

Doses [Gy] in organs at risk of all patients for 30 treatment fractions are demonstrated. Maximum shielding of the eyeballs resulted in mean doses in the optic nerves from 1.19 Gy to 46.18 Gy (max 63.44, min 0,88 Gy). The mean doses of the optic chiasm ranged from 5.09 Gy to 51.66 Gy (max 2.04 Gy, min 58.56).

The mean distance between planning target volume (PTV) and the nearest eyeball was 44.4mm (16.7–76.0mm).

Correlating the measurements with the MVCT data allowed a calculation of the longitudinal distance between the first registered light sensation and the orbital roof as a reference structure (Table 4); the maximum recorded distance was 56 mm; the average over all patients was 29 mm.

**Table 4:** Spatio-temporal distances of the first registered visual sensation.

Patient	Couch speed [cm/s]	Position 1 light sensation		Distance 1. light sensation to orbital roof	
		time [s]	distance [mm]	time [s]	distance [mm]
1	0.047	107	50.3	34.7	16.3
2	0.049	88.5	43.4	114.5	56.1
3	0.027	301	81.3	61.4	16.6
4	0.046	171	78.7	38.8	17.8
5	0.045	19.5	8.8	33.4	15
6	0.034	55.5	18.9	122.7	41.7
7	0.045	128.5	57.8	78.8	35.5
8	0.045	65	29.3	61.2	27.5
9	0.022	29	6.4	201.2	443
10	0.045	12	5.4	57.9	26.1
			Max	201.2	56.1
			Min	33.4	15
			Av	80.5	29.7
			Med	61.3	26.8
			σ	52.5	14.2

Patient specific spatio-temporal distances between the first registered light sensation and the bony structure orbital roof are shown. The interval between the beginning of the radiation and the first VSs ranges from 12 s (5.4 mm) to 301 s (81,3 mm). The distance between the first VSs and the orbital roof ranged from 33,4 s (15 mm) to 201,2 s (56,1 mm). All measured distances of the first beam inducing a VSs are more than ½ beam width away from the orbital roof.

## Discussion

The phenomenon of radiation-induced VSs has long been recognized [1, 3, 23]. Descriptions of the color and shape of the light phenomena are consistent in reports over the decades, but the phenomenon is still poorly understood. It is scientifically accepted that the blue light flashes are induced by direct irradiation of the retina, for example during space flights [4–18] in dark adapted eyes.

Observing VSs in a patient undergoing radiation treatment with a Tomotherapy unit caused us to further elucidate this finding in a group of patients who were treated for glioblastoma. The study tried to objectify subjective perceptions of patients during their radiation treatment. As patients were encluded, when they experienced VS during radiotherapy, it was not a randomized study, and we were not able to compare our results with a control group.

As visual sensations in patients undergoing tomotherapy treatment of brain tumors have neither been systematically reported nor evaluated, we had to compare our findings with VS occurring under other conditions.

In contrast to former reports on visual sensations, e.g. those occurring in astronauts, in our study VSs occurred regardless of whether the patient’s eyes were open or shut under roomlight conditions.

Correlation of VSs and beam parameters of the therapy unit revealed that the conditions under which VSs were recorded, were remarkably reproducible. This was the case not only within one treatment session but also between treatment sessions. Neither the position of the linac nor the beam path during occurrence of a VS was known by the patient.

Former publications discussing VSs lack information about the location of the light perception in the visual field. As illustrated in our results most beams triggering VS were located above the eye level (74460 / 97285). Assuming that radiation induces VSs by direct interaction with the retina, this should affect mainly upper retinal tissue, and consequently lead to phenomena in the lower visual field. However, our patients reported exactly the opposite, describing moving light sensations in the upper visual field. This means, nerval structures that usually carry information from lower retinal receptors to the visual cortex are responsible for the VS, further supporting the hypothesis of an extraretinal VSs origin.

The maximum vertical beam path distance to the orbital roof at the time of the first VS was 56 mm in our patients. Under such a condition radiation scattering causes only a very small dose reaching the intraorbital structures, as the dose fall-off with tomotherapy is steep: In a distance of 30mm from the treatment volume the dose is less than 1% of the prescribed dose [24]. With such small doses, induction of VSs seems improbable comparable to the results of Schardt et al. [25, 26].

In a second step of our analysis including all administered beams we were able to assign probabilities for VSs to occur in visual pathways.

Analyzing all beams, i.e. not excluding beams affecting the eyes, it turned out, that 20% of the beams affected the eyes, and only 10% of those induced VSs. On the contrary 22.3% of the beams passing structures as optic tract or the chiasma induced light perceptions. All analyzed Brodman areas showed a higher incidence of VSs than the eyes themselves. These results also support our thesis of extraretinal, retroorbital origin.

The dose exposition of optic nerves and optic chiasm shows a high variability with a wide range (from 2% to107% of the prescribed dose). There seems to be no obvious correlation between doses and the induction of visual sensations.

Our hypothesis seems to be supported by recent reports of VSs in two patients undergoing heavy ion therapy for skull base tumors [25, 26]. The authors described VSs even in the absence of detectable dose deposition in the eye. Cherenkov radiation was considered unlikely to account for VSs in these patients as the dose did not exceed the typical Cherenkov radiation threshold of 430 MeV/n; the authors did not offer a definitive explanation or point of origin for the VSs in these cases.

VSs occurred despite the fact that we maximally avoided direct beam paths through the orbits. Virtually shielding the eyes from the radiation by excluding all beams closer than 40 mm to the center of the nearest eyeball showed highest probability for being the origin of VSs (15%) for beams in a distance more than 40 mm occipital to the optic chiasm (Fig 3d).

VSs were unlikely to be due to radiation leakage: it has been demonstrated that because of the pronounced collimator shielding radiation leakage amounts to less than 0.05% of the administered dose [24]. If radiation leakage or scattering were responsible for VSs the phenomenon should be observed more frequently during conventional radiotherapy, which is associated with much more scattering and leakage of radiation.

The mechanism of radiation induced VSs by exciting extraocular intracerebral neuronal centers may be explained by molecular physiological studies. They suggest that radiation-induced increases in cell excitability are caused by depolarization processes in the neuronal tissue. The effects of radiation-induced free radicals (i.e. TNF-α) on microglial tissue and astrocytes should also be considered [27–30]. All these processes may play a role in VSs and should be investigated further.

In summary, this study analyzed 10 photon irradiated brain tumor patients who experienced VSs during Tomotherapy radiation treatment. We evaluated 97285 beams and over 5959 VSs registered during 175 treatment sessions. Highest probability for being the origin of VSs was shown for optic chiasm, optic tract and along the extraretinal visual pathways. To further localize anatomic regions in future, a larger number of patients as well as smaller target volumes, like in stereotactic irradiation, will be necessary.
